# The Value of CT Attenuation in Distinguishing Atypical Adenomatous Hyperplasia from Adenocarcinoma *in Situ*

**DOI:** 10.3779/j.issn.1009-3419.2013.11.03

**Published:** 2013-11-20

**Authors:** Binghu JIANG, Jichen WANG, Peng JIA, Meizhao LE

**Affiliations:** Department of Radiology, BenQ Medical Center, Nanjing 210019, China

**Keywords:** Atypical adenomatous hyperplasia, Adenocarcinoma *in situ*, Computed tomography

## Abstract

**Background and objective:**

Advances in high-resolution computed tomography (CT) scanning have increased the detection of small ground-glass opacity (GGO) nodules and also allowed such images to be investigated in detail. However, it is difficult to differentiate atypical adenomatous hyperplasia (AAH) from adenocarcinoma *in situ* (AIS) with CT, even at follow-up, because they share many similar CT manifestations. While AAH is thought to be a precursor or even an early-stage lesion of lung adenocarcinoma, and the stepwise progression from AAH to AIS is thought to be reasonable. Therefore, the hypothesis that the attenuation of GGO is increased gradually from AAH to AIS is proposed. The aim of this study was to distinguish AAH from AIS with CT attenuation in patients with pure GGO nodules.

**Methods:**

Between January 2010 and December 2012, the CT findings in terms of the greatest diameter and mean CT attenuation (HU) were reviewed and correlated with pathology in 56 patients with AAH (*n*=21) and non-mucinous AIS (*n*=38) by two independent observers. All the 59 lesions were pure GGO nodules with size of 2 cm or smaller. To determine variability of measuring CT attenuation, we calculated the 95% confidence interval (CI) for the limits of agreement by using Bland-Altman analysis. Student t test was used to compare AAH and AIS in terms of diameter and CT attenuation. And receiver operating characteristic (ROC) curve was used to determine the optimal cut-off value of mean CT attenuation for differentiating AAH from AIS and obtain the diagnostic value. Two-tailed *P* value of less than 0.05 was considered to be significant.

**Results:**

For the manually measured CT attenuation, the 95%CI for the limits of agreement was -40 HU, 50 HU for inter-observer variability. Although there was significant difference in nodule diameter between AAH and AIS (*P*=0.046), the overlap was considerable. The mean CT attenuation was (-718±53) HU (95%CI: -822, -604) for AAH, which was significantly smaller than (-600±35) HU (95%CI: -669, -531) for AIS (*P*=0.013). The area under curve (AUC) from ROC was 0.903 for differentiating AAH from AIS, and the cut-off value of -632 HU was optimal for differentiation between AAH and AIS, with sensitivity of 0.79, specificity of 0.95, and accuracy of 0.85.

**Conclusion:**

The mean CT attenuation can help the radiological differentiation between AAH and AIS.

## Introduction

The popularization of computed tomography (CT) in clinical practice and the introduction of mass screening for early lung cancer with the use of CT have increased the frequency of findings of subtle nodules or nodular ground-glass opacity (GGO). GGO is defined as hazy increased opacity of lung, with preservation of bronchial and vascular margins^[1]^. For lesions with GGO, a number of differential diagnoses are possible, including benign conditions, malignancies such as adenocarcinoma of the lung, especially adenocarcinoma *in situ* (AIS), as well as putative precursors such as atypical adenomatous hyperplasia (AAH)^[2]^. The persistence of nodular GGO over time may be strongly suggestive of an early-stage malignancy, especially if the lesion increases in size or attenuation.

Differentiation between AAH and AIS is important because that surgery is needed for AIS, whereas AAH can be safely just followed by CT^[3]^. However, it is generally difficult because they share many CT features and may occur concurrently^[4]^. An awareness of the significance of CT attenuation in assessing GGO has been reported recently ^[5-8]^. The aim of this study was to distinguish AAH from AIS on the basis of International association for the study of lung cancer/American thoracic society/European respiratory society (IASLC/ATS/ERS) classification of lung adenocarcinoma^[9]^ with mean CT attenuation.

## Materials and Methods

This retrospective study was approved by our institutional review board and written informed consent was obtained for the use of thin-section CT from all participants.

### Patients

Between January 2010 and December 2012, 36 patients (17 men and 19 women, mean age 63.5 years ±8.7 SD) pathologically diagnosed with AIS, who underwent thin-section CT examinations before surgical resection at our hospital. During the same period, 20 patients (10 men and 10 women, mean age 62.5 years ±7.2 SD) with AAH underwent thin-section CT examinations before thoracoscopic biopsy. The interval between thin-section CT and surgery was 16 days ±14 SD with range of 1 to 35 days. One of the 20 patients with AAH had two lesions, and two of the 36 patients with AIS had two lesions, respectively. All of the 59 lesions were pure GGO with size of ≤2 cm.

### CT scanning

Patients underwent imaging with a 16-slice CT scanner (Aquilion 16; Toshiba, Tokyo, Japan) in helical mode with 16×0.5 mm collimation and 0.5 seconds per rotation. Scans were obtained with the patient at full inspiration, without previous training. No intravenous contrast material was injected. Exposure settings were 120-150 mAs at 120 kVp. Axial images of 1.0 mm thickness with 0.5 mm spacing were reconstructed with 512×512 matrix by using high-frequency algorithm and the smallest field of view that included both lungs. Air calibration was conducted every morning before CT scans.

### Measurements of CT number

Without knowledge of pathology, two observers (P.J. and B.J., with 5-year and 4-year experience in chest radiology, respectively) independently measured each nodule with the aid of a monitor (Toshiba Medical Systems).

Each nodule was measured with a fixed lung window setting (level, -600 HU; width, 1, 200 HU) in terms of diameter (cm) and mean CT attenuation (HU). We determined the greatest diameter by using the electronic calipers function on the axial image on which the GGO had the maximum dimension. For the measurements of mean CT attenuation, vessels and bronchi within GGO were excluded from the regions of interest.

### Pathology

The specimens were stained with hematoxylin-eosin. All slides were reviewed by a pathologist (M.L., with 30-year experience in surgical pathology). AAH is a localized, small proliferation of mildly to moderately atypical type Ⅱ pneumocytes or Clara cells lining alveolar walls and respiratory bronchioles. Gaps are usually seen between the cells, which consist of rounded, cuboidal, low columnar or "peg" cells with round to oval nuclei (**Fig 1**). AIS, a localized small adenocarcinoma with growth restricted to neoplastic cells along preexisting alveolar structures (lepidic growth), there is a lack of stromal, vascular, or pleural invasion (**Fig 2**) ^[9]^.

**1:**
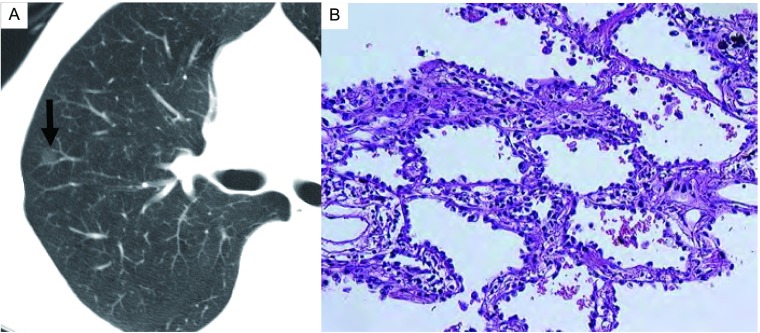
Atypical adenomatous hyperplasia (AAH) in a 58-year-old woman. A: Thinsection CT image shows faint ground-glass opacit y (arrow). B: Photomicrograph of histologic specimen shows small proliferation of mildly to moderately atypical cells lining alveolar walls (Hematoxylin-eosin stain; original magnification, ×20). CT: computed tomography.

**2:**
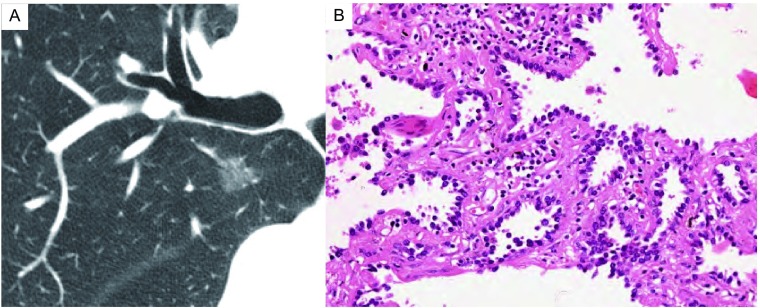
Adenocarcinoma *in situ* (AIS) in a 67-year-old man. A: Thin-section CT image shows ground-glass opacit y (arrow). B: Photomicrograph of histologic specimen shows neoplastic cells lining preexisting alveolar structures without stromal, vascular, or pleural invasion (Hematoxylin-eosin stain; original magnification, ×20).

### Statistical analysis

To determine inter-observer variability for measurements of mean CT attenuation, we calculated the 95% confidence interval (CI) for the limits of agreement by using Bland-Altman analysis^[10]^. The data of mean CT attenuation was compared between AAH and AIS by Student t test. The receiver operating characteristic (ROC) curve was performed to determine the optimal cut-off value of mean CT attenuation and diagnostic value for differentiating AAH from AIS. Two-tailed *P* value of less than 0.05 was considered to be significant. All of the statistical calculations were performed with SPSS software (PASW Statistics 18; SPSS Inc., Chicago, IL, USA).

## Results

All of the 38 AIS were non-mucinous and the pathologic TNM stage was T1N0M0. There was no significant difference between patients with AAH and AIS in terms of age or sex (*P* > 0.05). The average of diameters was 0.7 cm ±0.4 SD (range: 0.3 to 1.2 cm) for AAH, which was significantly smaller than 1.2 cm ±0.6 SD (range: 0.6 to 2.0 cm) for AIS (*P*=0.046) (**Tab 1**).

**1:** Characteristics of patients with atypical adenomatous hyperplasia (AAH) and adenocarcinoma *in situ* (AIS).

Variable	AAH	AIS	*P*
Number of lesions	21	38	
Sex (men/women)	10/10	17/19	0.842
Age (Mean±SD) (year)	62.5±7.2	63.5±8.7	0.796
Tumor size (Mean±SD) (cm)	0.7±0.4	1.2±0.6	0.046

The Bland-Altman plots showed the limits of agreement was 5 HU ±23 SD with 95%CI of -40 HU to 50 HU for inter-observer variability of CT attenuation (**Fig 3**). The mean CT attenuation was -718 HU ±53 SD (95%CI: -822--604) for AAH with range of -819 HU to -615 HU, which was significantly smaller than -600 HU ±35 SD (95%CI: -669--531) for AIS with range of -681 HU to -524 HU (*P*=0.013) (**Fig 4**).

**3:**
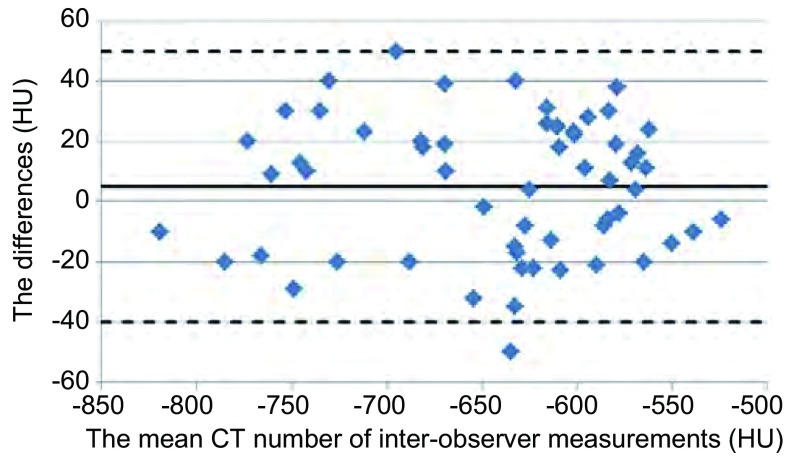
Bland-Altman plots show inter-observer variability for mean CT attenuation (HU)

**4:**
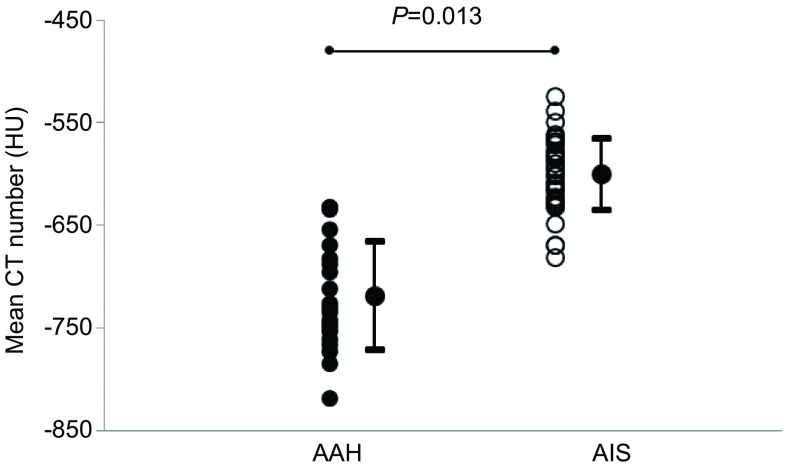
Comparison of mean CT attenuation (HU) according to histologic type of tumor. The mean CT attenuation becomes significantly greater in the progression from AAH (-718±53 HU) to AIS (-600±35 HU) (*P*=0.013). AAH: atypical adenomatous hyperplasia; AIS: adenocarcinoma *in situ*.

The cut-off value of -632 HU with area under curve (AUC) of 0.903 was optimal for differentiation between AAH and AIS, with sensitivity of 0.79, specificity of 0.95, positive predictive value (PPV) of 0.97, negative predictive value (NPV) of 0.71, and the accuracy of 0.85, respectively (**Fig 5**).

**5:**
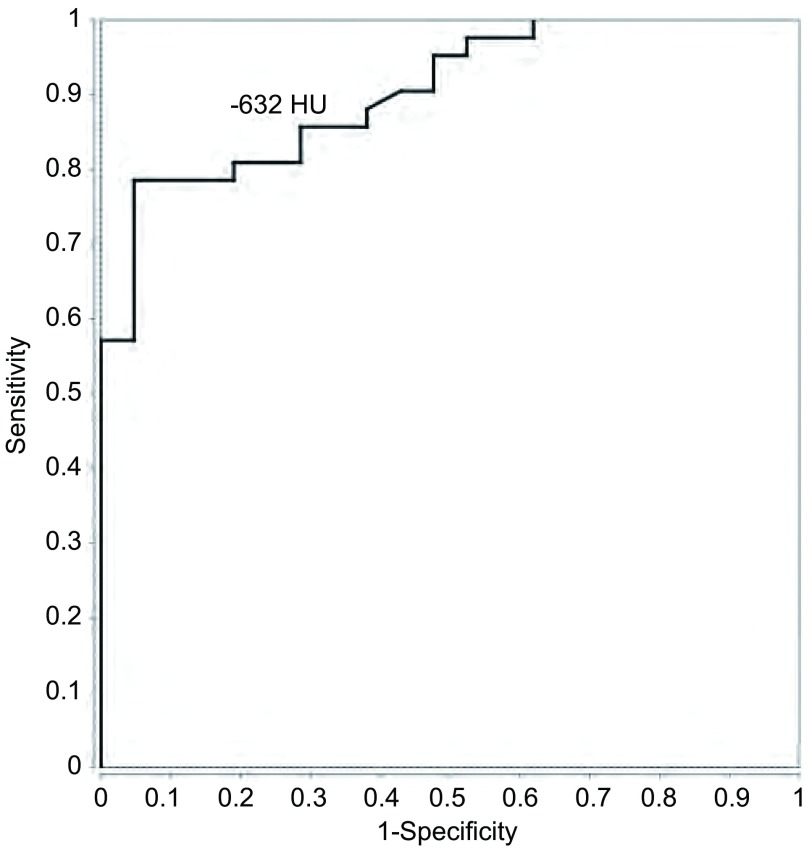
ROC curve of the mean CT attenuation for differentiation between atypical adenomatous hyperplasia (AAH) and adenocarcinoma *in situ* (AIS), showing the optimal cut-off value is -632 HU.

## Discussion

In the current study, mean CT attenuation of pure GGO was confirmed to be useful in differentiating AAH from AIS with the cut-off value of -632 HU. Although there was a significant difference between AAH and AIS in terms of size, the overlap was considerable.

Pulmonary GGO has been reported to be caused by partial filling of airspaces, interstitial thickening (due to fluid, cells, or fibrosis), partial collapse of alveoli, increased capillary blood volume, or a combination of these, the common factor being the partial displacement of air^[11]^. GGO is therefore a combination of both soft tissue and air-contained spaces. Thus, the attenuation of the GGO nodule at CT is a combination of the attenuation of soft tissue and air. It is reasonable to assume that changes in the attenuation values for the GGO nodule can vary considerably with corresponding changes in the proportion of soft tissue. As we know, AAH is a localized, small (usually ≤5 mm) proliferation of atypical type Ⅱ pneumocytes or Clara cells lining the alveolar walls and respiratory bronchioles, whereas AIS (one of the lesions formerly known as BAC) is a localized small (≤3 cm) adenocarcinoma in which growth of neoplastic cells is restricted to preexisting alveolar structures (lepidic growth) that lack stromal, vascular, or pleural invasion^[9]^. Thus, AAH usually has more air spaces and fewer cellular components than AIS, so that the former usually has significantly lower CT attenuation than the latter.

Nomori *et al*^[5-7]^ reported that the peak or mean CT attenuation on the histogram can help the radiologic differentiation between AAH and bronchioloalveolar carcinoma (BAC). In addition, two peaks on the CT number histogram can rule out AAH, and the mean CT attenuation is the optimal CT attenuation for differentiating BAC from adenocarcinoma of the lung. However, the term BAC has been recommended to be of discontinuing use by the IASLC/ATS/ERS^[8]^ because of its broad spectrum of tumors including solitary small noninvasive peripheral lung tumors with a 100% 5-year survival, invasive adenocarcinomas with minimal invasion that have approximately 100% 5-year survival, mixed subtype invasive adenocarcinomas, mucinous and non-mucinous subtypes of tumors formerly known as BAC, and widespread advanced disease with a very low survival rate. In addition, CT attenuation on the histogram can be performed only with computer aided design (CAD), which is of limited application in daily clinical practice, whereas the mean CT attenuation is easy to obtain in any CT model.

However, there were limitations to our study. First, the subjects were limited in pure GGO lesions with relatively small number of patients. Second, our study was a retrospective design, possibly leading to a sampling bias. Third, CT attenuation was affected by CT scanner, voltage of X-ray exposure, and reconstruction algorithm, the exposure settings varied between 120 mAs and 150 mAs in our study, which might shadow our results.

In conclusion, AAH can be distinguished from AIS on the basis of the mean CT attenuation. Pulmonary pure GGO nodules that are small in diameter (usually ≤1.0 cm) and less than -632 HU on the mean CT attenuation can be followed by CT rather than surgical biopsy or resection. According to the tumor doubling time^[12]^, a follow-up of two or three years would be safe and enough to confirm whether or not the lesions are AAH.

## Conflict of interest

The authors report no conflicts of interest.
